# RGMa Nuclear Localization in Skeletal Muscle Cells Reveals a Novel Role in Cell Viability and Proliferation

**DOI:** 10.3390/cells15020161

**Published:** 2026-01-15

**Authors:** Cristhian David Andrade Alfaro, Julia Meireles Nogueira, Christhiam Douglas Caetano Ribeiro, Kirsty Ximena Noboa Carrasco, Ana Luísa Cremonese Lubiana, Ana Maria Alvarenga Fagundes, Natália Paloma Vieira de Souza, Victor Rodrigues Santos, Carolina Cattoni Koh, Walderez Ornelas Dutra, Erika Cristina Jorge

**Affiliations:** Departamento de Morfologia, Instituto de Ciências Biológicas, Universidade Federal de Minas Gerais, Av. Pres. Antônio Carlos, 6627, Belo Horizonte 31270-901, MG, Brazil; cd.andrade.alfaro@gmail.com (C.D.A.A.); jumeirelesn@gmail.com (J.M.N.); ccribeiro@aluno.fiocruz.br (C.D.C.R.); kxnoboa@gmail.com (K.X.N.C.); luisalubiana@gmail.com (A.L.C.L.); anamariafagundes11@gmail.com (A.M.A.F.); nahsouza027@gmail.com (N.P.V.d.S.); neurovrs@gmail.com (V.R.S.); carolinakoh@gmail.com (C.C.K.); waldutra@gmail.com (W.O.D.)

**Keywords:** primary skeletal muscle cells, repulsive guidance molecule, subcellular compartment, protein translocation

## Abstract

The Repulsive Guidance Molecule a (RGMa) is a multifunctional GPI-anchored protein localized in the sarcolemma and sarcoplasm of the adult skeletal muscle cell. Our research group showed that RGMa overexpression can promote myoblast fusion and induce hypertrophic muscle fibers during in vitro differentiation. Here, we report that RGMa is expressed in primary skeletal muscle cells cultured in vitro, showing a nuclear localization, revealed by immunostaining with an antibody targeting its C-terminal region (C-RGMa). While RGMa was detected in the nuclei, its canonical receptor, Neogenin, was predominantly found in the perinuclear region. Nuclear RGMa was absent in Neogenin-knockdown cells, suggesting that Neogenin mediates its nuclear transport. Functional assays suggested that RGMa promotes primary skeletal muscle cell viability and proliferation and supports their myogenic commitment. These findings reveal a previously unrecognized nuclear function of RGMa–Neogenin signaling and provide new insights into the regulation of skeletal muscle cell behavior in vitro.

## 1. Introduction

Different signaling pathways control skeletal muscle cell maintenance and proliferation. Our research group has been studying the molecular mechanisms induced by the RGMa-Neogenin signaling pathway during skeletal muscle development and regeneration. Repulsive Guidance Molecule a (RGMa) is a glycosylphosphatidylinositol (GPI) anchor protein, first described playing roles as an axon guidance molecule [1]. It binds to the type I transmembrane protein Neogenin and mediates axon guidance and neuron survival through the FAK-RhoA signaling pathway [2,3]. RGMa can also signal as a BMP coreceptor since RGMa and the BMP type I receptor ectodomain (BMPR1A) share the same binding site on the BMP ligand [4].

RGMa-Neogenin action domains expanded to non-neuronal tissues, including its association with skeletal muscle. RGMa transcripts were found expressed in the precursor locations of satellite cells, in the somites of chicken embryos, where their expression colocalized with Pax7 [5]. In the adult skeletal muscle cell, RGMa was in the sarcoplasm and sarcolemma of some fibers [6]. As a GPI-anchor protein, RGMa is expected to be found on the cell surface [7]. RGMa overexpression (cloned in an expression plasmid) or treatment (recombinant protein) in immortalized C2C12 lineage or in primary skeletal muscle cells induced an increase in the fusion index and in myotubes with increased size and length, suggesting that RGMa can induce hyperplasia and hypertrophy in skeletal muscle cells [6,8]. RGMa effects on cell size and fusion were found to be mediated via Neogenin receptor, and these phenotypes were improved when the BMP signaling pathway was inhibited [8]. Additionally, through transcriptome analysis of C2C12 cells treated with recombinant RGMa protein, it was found that both Neogenin and BMP pathways are active in skeletal muscle cells, inducing phenotypes that control cell size and modulating various signaling pathways linked to muscle hypertrophy [9].

In this study, we investigated whether RGMa is expressed in primary skeletal muscle cells and its possible functions. We found that RGMa is expressed in the nuclei of primary skeletal muscle cells, while Neogenin was found in the perinuclear region of these cells. RGMa-Neogenin nuclear localization is a significant finding since it is a GPI-anchor protein and a transmembrane receptor, respectively. Furthermore, functional studies performed in vitro suggested possible new roles for this axon guidance molecule in skeletal muscle cell viability, proliferation, and differentiation.

## 2. Materials and Methods

### 2.1. Animals

Wild-type male C57BL/6J mice aged 6–8 weeks were obtained from the Central Animal Facility (CEBIO) at the Federal University of Minas Gerais (UFMG). All procedures involving animals were conducted in accordance with ethical guidelines and were approved by the Ethics Committee on the Use of Animals (CEUA/UFMG; protocol numbers 366/2019 and 198/2025). Mice were housed under standard conditions in the animal facility of the Department of Morphology at UFMG, maintained at a constant temperature of 24 °C under a 12 h light/12 h dark cycle, with ad libitum access to food and water.

### 2.2. Primary Skeletal Muscle Cells Isolation from EDL Myofibers

Primary skeletal muscle cells were isolated from single myofibers of the extensor digitorum longus (EDL) muscle from three male C57BL/6J mice, as previously described [10,11,12]. Briefly, EDL muscles were dissected tendon-to-tendon and enzymatically digested in 0.2% type I collagenase (Gibco, #17100-017, Waltham, MA, USA) diluted in high-glucose Dulbecco’s Modified Eagle Medium (DMEM; Gibco, #12100046, Waltham, MA, USA) for 40–50 min at 37 °C with gentle shaking every 10 min. Following digestion, muscles were transferred to a pre-warmed growth medium (GM) composed of high-glucose DMEM supplemented with 20% fetal bovine serum (FBS; Gibco, #12657-029, Waltham, MA, USA) and 1% antibiotic-antimycotic solution (Gibco, #15240-062, Waltham, MA, USA). Individual myofibers were released by gentle trituration using a plastic pipette previously coated with horse serum (HS, Gibco, #16050122, Waltham, MA, USA). Live, intact myofibers were transferred first to a pre-warmed dish coated with HS, and subsequently to a culture flask containing fresh GM. Myofibers were maintained at 37 °C in a humidified incubator with 5% CO_2_. On the fourth day post-isolation, the growth medium was removed, and the flasks were gently washed with phosphate-buffered saline (PBS) to dislodge the myofibers, leaving the adherent primary skeletal muscle cells attached to the flask surface. Fresh GM was added, and cultures were maintained until cells reached approximately 80% confluence. For downstream analyses, cells were seeded at a density of 2 × 10^4^ cells/mL per well and maintained at 37 °C with 5% CO_2_. The growth medium was refreshed every two days.

### 2.3. Immunofluorescence Staining

Single myofibers and primary skeletal muscle cells were fixed with 4% paraformaldehyde (PFA, Sigma-Aldrich, #P6148, Burlington, VT, USA), permeabilized with 0.2% Tween-20 (Thermo Fisher Scientific, #85113, Waltham, MA, USA) in PBS (PBST) at 37 °C, and blocked with 5% bovine serum albumin (BSA, Sigma-Aldrich #A2153) in PBS for 1 h at room temperature (RT). Cells were then incubated overnight at 4 °C with the following primary antibodies: anti-RGMa (1:100, Thermo Fisher Scientific, #PA5-115836, Waltham, MA, USA), anti-Pax7 (1:50, DSHB), anti-MyoD (1:50, Thermo Fisher Scientific, #MA5-12902, Waltham, MA, USA), and anti-Neogenin (1:100, Thermo Fisher Scientific, #PA-55695, Waltham, MA, USA). Control samples were incubated with blocking solution only, without primary antibodies. After washing with PBS, cells were incubated with secondary antibodies (1:500, Thermo Fisher Scientific, Alexa Fluor 488, #A11008, Waltham, MA, USA or 1:500, Thermo Fisher Scientific, Alexa Fluor 555, #A21426, Waltham, MA, USA) for 1 h at RT. Nuclei were counterstained with DAPI (4′,6-diamidino-2-phenylindole; Thermo Fisher Scientific, #D1306, Waltham, MA, USA). Fluorescence images were acquired using a Zeiss inverted fluorescence microscope (Jena, Germany), and image processing was performed with Zeiss ZEN Blue 3.8 software.

### 2.4. Confocal Microscopy

For high-resolution confocal imaging, 0.17 mm glass coverslips were pre-treated by overnight incubation in 10% hydrochloric acid (*v*/*v*), followed by thorough rinsing. Prior to cell seeding, coverslips were placed in culture wells and pre-incubated with growth medium (GM) to enhance cell adhesion. The immunofluorescence protocol described above was then applied to cells grown on the coverslips. After staining, coverslips were mounted with Fluoromount-G (Invitrogen, #00-4958-02, Waltham, MA, USA) to minimize refractive index mismatch and improve imaging quality. Confocal images were acquired using a Zeiss LSM 880 Airyscan microscope. Z-stack imaging was performed to confirm the subcellular localization of RGMa and Neogenin in primary skeletal muscle cells.

### 2.5. Immunocytochemical Peroxidase Staining

Cells were fixed with 4% paraformaldehyde (PFA, Sigma-Aldrich, #P6148, Burlington, VT, USA), washed with PBS, and blocked using a 1% hydrogen peroxide solution in PBS to quench endogenous peroxidase activity. After three 5 min washes with PBS, samples were permeabilized with PBST and subsequently blocked with 5% bovine serum albumin (BSA, Sigma-Aldrich #A2153, Burlington, VT, USA) in PBS. Cells were then incubated overnight at 4 °C with anti-RGMa (1:100, Thermo Fisher Scientific, #PA5-115836, Waltham, MA, USA) and anti-Neogenin (1:100, Thermo Fisher Scientific, #PA-55695, Waltham, MA, USA) primary antibodies. After PBS washes, the EnVision+ System-HRP Dual Link (Dako, anti-mouse and anti-rabbit, #106816-004, Glostrup, Dinamarca) secondary antibody was applied and incubated for 1 h at room temperature. Signal detection was performed using 3,3′-diaminobenzidine (DAB; Biotium #30015, Fremont, CA, USA), and nuclei were counterstained with hematoxylin. Following staining, samples were post-fixed with 4% PFA, and images were acquired using an Olympus BX41 microscope (Shinjuku City, Japan) equipped with a Motic AE31 image capture system.

### 2.6. Western Blot Assay

Primary skeletal muscle cells were cultivated until they reached 80% confluency and homogenized and lysed in RIPA Buffer supplemented with protease inhibitor cocktail. Obtained lysates were kept on ice for 30 min, centrifuged, and stored at −80 °C. Protein concentration was determined using Bradford’s assay (Biorad, Shinagawa City, Japan). Twenty micrograms of each protein was subjected to 10/12% SDS-PAGE and Western blot analysis and probed with the antibody against RGMa (1:100; Thermo Fisher, PA5-115836). As a secondary antibody, Dako EnVision dual link System-HRP (DAB+) was used. Images were captured using the UVP Geldoc imaging system (Thermo Fisher).

### 2.7. Subcellular Localization Prediction of RGMa and Neogenin

The amino acid sequences of Neogenin (accession number AAH54540.1) and RGMa (accession number AAH59072.1) were retrieved from the NCBI database. PSORT II software WWW version was used to predict potential nuclear localization signals (NLS) and to assess the subcellular localization profiles of both proteins.

### 2.8. siRNA Transfection

After 24 h of seeding and upon reaching approximately 80% confluence, primary skeletal muscle cells were transfected with 5 μM of Silencer^®^ Select Negative Control siRNA (Ambion, #4390843, Austin, TX, USA), Neogenin (Neo1) Silencer^®^ Select Pre-designed siRNA (Ambion, #4390771 ID: s704077, Austin, TX, USA), or RGMa Silencer^®^ Select Pre-designed siRNA (Ambion, #4390771 ID: s110205, Austin, TX, USA). siRNA–lipid complexes were prepared using Lipofectamine™ 3000 (Thermo Fisher Scientific, #L3000-015, Waltham, MA, USA) and Opti-MEM^®^ Reduced Serum Medium (Thermo Fisher Scientific, #31985062, Waltham, MA, USA), following the manufacturer’s protocol. Cells were incubated for 48 h to ensure optimal transfection efficiency.

### 2.9. Cell Viability Assay

Cell viability following gene knockdown was assessed using the MTT assay (3-(4,5-dimethylthiazol-2-yl)-2,5-diphenyltetrazolium bromide; Thermo Fisher Scientific, #M6494, Waltham, MA, USA), according to the manufacturer’s instructions. Briefly, MTT was diluted in culture medium (1:9, MTT:medium), added to each well, and incubated for 2 h at 37 °C in a 5% CO_2_ atmosphere. The solution was then removed, and 200 μL of acidified isopropanol (prepared with 100 mL isopropanol and 134 μL hydrochloric acid) was added to each well to solubilize the formazan crystals. Aliquots (50 μL/well) were transferred to a 96-well plate in triplicate, and absorbance was measured at 595 nm using a BioTek^®^ ELX800 microplate reader. All experiments were performed in triplicate.

### 2.10. Flow Cytometry

Primary skeletal muscle cells were detached from culture plates and fixed with 2% paraformaldehyde (PFA, Sigma-Aldrich, #P6148, Burlington, VT, USA) in PBS. Cells were then permeabilized using 0.01% Tween-20 (ThermoFisher, #85113, Waltham, MA, USA) in PBS and incubated overnight at 4 °C with an anti-Ki67 antibody (Novus Biologicals, 1:50, NB500-170, Cetennial, CO, USA). After washing with permeabilization buffer, cells were incubated with an Alexa Fluor 488-conjugated secondary antibody (1:500, Thermo Fisher Scientific, Alexa Fluor 488, #A11008, Waltham, MA, USA). Finally, cells were resuspended in PBS for analysis. A total of four experimental conditions were prepared: (i) unstained cells, (ii) cells incubated with secondary antibody only, (iii) cells transfected with control siRNA, and (iv) cells transfected with RGMa siRNA. All samples were analyzed in triplicate. Data acquisition was performed using a FACSCanto II flow cytometer (Becton Dickinson, San Jose, CA, USA), and data were processed using FlowJo software (version 10.10).

### 2.11. qPCR

Total RNA was isolated from primary skeletal muscle cells 48 h post-transfection using TRI Reagent (Sigma-Aldrich, #T9424, Burlington, VT, USA), following the manufacturer’s protocol. RNA integrity was verified by electrophoresis, and concentration and purity were determined using a NanoDrop 2000 spectrophotometer (Thermo Fisher Scientific). A total of 1 μg of RNA was reverse-transcribed into cDNA using the iScript™ cDNA Synthesis Kit (Bio-Rad, #1708891, Hercules, CA, USA). Quantitative PCR was performed using the Rotor-Gene Q RT-PCR system (Qiagen, Venlo, The Netherlands). Each 10 μL reaction contained 1× iTaq™ Universal SYBR^®^ Green Supermix (Bio-Rad, #1725121), 0.4–0.6 μM of each primer, and 1 μL of cDNA (diluted 1:10). Reactions were run in triplicate under the following cycling conditions: initial denaturation at 95 °C for 2 min, followed by 45 cycles of 94 °C for 15 s, 60–62 °C for 15 s, and 72 °C for 20 s; with a final extension step at 72 °C for 5 min. A dissociation (melting) curve analysis was included to confirm amplification specificity. qPCR data were analyzed using Rotor-Gene 8 software with a threshold set at 0.05. Further statistical analysis and graph generation were performed using R software v.4.5.2 and GraphPad Prism 9. Primer sequences used in this study were as follows: GAPDH: AGGTCGGTGTGAACGGATTTG/TGTAGACCATGTAGTTGAGGTCA, RGMa: CCACATCAGGAAGGCAGAAG/GCGTAGCACTGGGTAGGAAG, Neogenin: TCCGTTTTATTTTCTGGTGGA/CGTCAGGCTTATTGTGTTTGG, Myf5: ACTGGCGTGTCTCCTCTCT/TCAAACTGGTCCCCAAACTC, MyoD: GTGGCAGCGAGCACTACA/GACACAGCCGCACTCTTC, Myogenin (MyoG): TGAGAGAGAAGGGGGAGGAG/CGGTATCATCAGCACAGGAG, Myh3: GCAAAGACCCGTGACTTCAC/GCATGTGGAAAAGTGATA, Runx2: GCCACCACTCACTACCACAC/CAGCGTCAACACCATCATTC, Osterix: GAGGAAGAAGCCCATTCACA/TTGGAGCAGAGCAGACAGG and Pax7: TACTGCCCACCCACCTACA/AGCGGAGTGTTCCCCAAG.

### 2.12. Statistical Analysis

Statistical analyses were performed using GraphPad Prism software (version 9.0). Data normality was assessed using the Shapiro–Wilk test. For comparisons between two groups, a two-tailed Student’s *t*-test was applied. In experiments involving three or more groups within a single categorical variable, one-way ANOVA followed by Tukey’s post hoc test was used. Data are presented as mean ± standard deviation (SD). All experiments were independently repeated at least three times. Results were considered statistically significant at *p* < 0.05 (*), *p* < 0.01 (**), and *p* < 0.001 (***).

## 3. Results

### 3.1. Immunolocalization of RGMa and Neogenin in Primary Skeletal Muscle Cells

**Figure 1:**
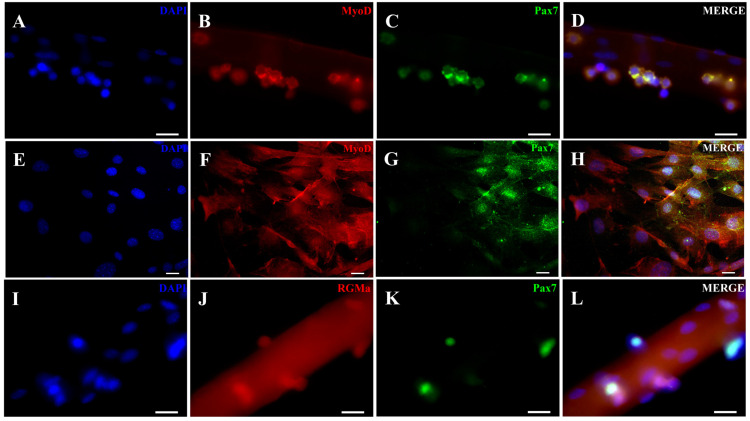
RGMa immunolocalization in mice isolated myofibers and primary skeletal muscle cells. (**A**) Myonuclei present in a freshly isolated myofiber stained with DAPI (blue). (**B**) Immunofluorescence staining for MyoD (red) and (**C**) for Pax7 (green) in the same isolated myofiber. (**D**) Merged image of panels (**A**–**C**), showing cells stained for both Pax7/MyoD markers. (**E**) Nuclei of primary skeletal muscle cells stained with DAPI (blue). (**F**) MyoD immunostaining and (**G**) Pax7 immunostaining in these cells. (**H**) Merged image of panels (**E**–**H**). (**I**) Myonuclei present in a freshly isolated myofiber stained with DAPI (blue). (**J**) Immunofluorescence staining for RGMa (red) and (**K**) Pax7 (green) in the same isolated myofiber. (**L**) Merged image of panels (**I**–**L**). Scale bars: 20 μm.

**Figure 2:**
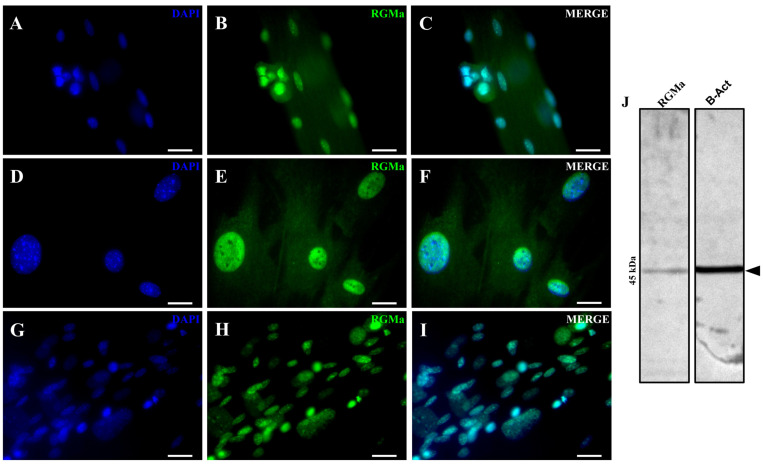
RGMa expression in the myonuclei of cells in an isolated myofiber and in primary skeletal muscle cells in culture. (**A**) DAPI staining (blue) of nuclei in a myofiber cluster. (**B**) RGMa immunostaining (green) in the same sample. (**C**) Merged image of (**A**,**B**). (**D**) DAPI staining (blue) of primary skeletal muscle cells that migrated to the culture surface. (**E**) RGMa expression (green). (**F**) Merged image of (**D**,**E**). (**G**) DAPI staining (blue) of primary skeletal muscle cells induced to differentiate. (**H**) RGMa immunostaining (green) in aligned skeletal muscle cells. (**I**) Merged image of (**G**,**H**). (**J**) Western blot analysis indicates the presence of a 45 kDa RGMa fragment in the pool of primary skeletal muscle cells. Scale bar: 20 μm.

**Figure 3:**
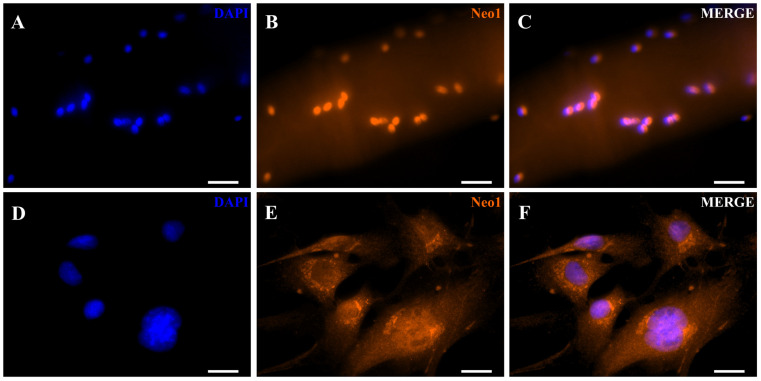
Neogenin expression in myonuclei of an isolated myofiber and in primary skeletal muscle cells. (**A**) DAPI staining (blue) of nuclei in an isolated myofiber. (**B**) Neogenin immunostaining (red) in the same sample. (**C**) Merged image of (**A**,**B**). (**D**) DAPI staining (blue) of primary skeletal muscle cells (**E**) Neogenin expression (red) in these cells. (**F**) Merged image of (**D**,**E**). Scale bar: 20 μm.

**Figure 4:**
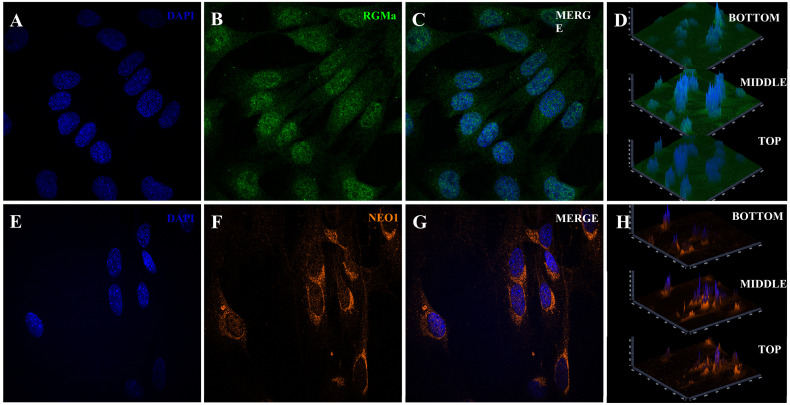
Z-stack confocal analysis of RGMa and Neogenin in primary skeletal muscle cells. (**A**–**D**) RGMa detection by Z-stack confocal microscopy. (**A**) DAPI (blue) staining. (**B**) RGMa (green) expression in the cell nuclei. (**C**) merge of (**A**–**C**). (**D**) Z-stack slices showing RGMa distribution at the bottom, middle, and top (**D**) of the cell. Stronger nuclear RGMa signals are observed in the middle slice. (**E**–**H**) Neogenin detection by Z-stack confocal microscopy. (**E**) DAPI (blue) staining. (**F**) Neogenin (orange) staining in these primary skeletal muscle cells. (**G**) merge of (**E**–**G**). (**H**) Z-stack slices showing Neogenin distribution at the bottom, middle, and top of the cell. Neogenin is primarily found in the perinuclear region, with additional signal in the cytoplasm and nuclei. 63× objective.

We first investigated whether RGMa is expressed in activated myoblasts derived from single isolated myofibers. The identity of these cells was confirmed by the expression of Pax7 and MyoD (Figure 1A–D), as well as in their progeny, cells that had migrated from the myofibers to the culture surface (referred to as primary skeletal muscle cells, Figure 1E–H). We then examined the expression of RGMa and Neogenin in these cells (Figure 1I–L, Figure 2 and Figure 3). Nuclear immunolocalization of RGMa was consistently observed in all myonuclei along the myofiber (Figure 1I–L), as well as in the nuclei of all primary skeletal muscle cells (Figure 2). RGMa expression persisted in the nuclei of differentiated cells after 5 days in differentiation medium (Figure 2G–I) and in immortalized myoblasts of the C2C12 lineage (Supplementary Figure S1). Western blot analysis confirmed the presence of a 45 kDa fragment of RGMa in the pool of skeletal muscle cells in the expected size (Figure 2J). Neogenin was also detected in all myonuclei within the isolated myofiber (Figure 3A–C). In contrast, in the primary skeletal muscle cells, Neogenin immunolocalization was predominantly perinuclear (Figure 3D–F). This distribution pattern was further confirmed by immunohistochemical staining (Supplementary Figure S2). To further assess the subcellular distribution of these proteins in primary skeletal muscle cells, high-resolution Z-stack images were acquired using the Confocal Spinning Disk Yokogawa—3i microscope (Figure 4). RGMa signals were clearly detected within the nuclei (Figure 4A–C). Z-stack analysis revealed weak RGMa expression at the apical and basal regions of the nucleus, with stronger signal intensity in intermediate planes (Figure 4D). Similarly, Neogenin was observed primarily in the perinuclear region but was also detected throughout the cytoplasm and within nuclei (Figure 4E–G). Neogenin intensity varied across Z-stack planes, displaying weaker signals in the basal region, moderate intensity in the apical region, and stronger expression in intermediate planes (Figure 4H).

### 3.2. Amino Acid Sequence Analysis of RGMa and Neogenin

To explore potential determinants of subcellular localization, we analyzed RGMa and Neogenin amino acid sequences using the PSORTII prediction tool (Table 1). RGMa did not present any predicted nuclear localization signals (NLS). The software indicated a predominant cytoplasmic localization (70.6%) and identified two dileucine motifs, one at the N-terminus (LL37) and another at the C-terminus (LL381), which are commonly found in membrane-associated proteins targeted to endosomes or lysosomes via clathrin-coated vesicles. In contrast, Neogenin amino acid sequence showed a high nuclear localization probability (89%) and did not contain dileucine motifs (Table 1). These findings indicate that PSORTII-based predictions do not fully account for the observed localization patterns of RGMa and Neogenin in MuSC and their myoblast progeny.

### 3.3. RGMa Nuclear Expression Depends on Neogenin in Primary Skeletal Muscle Cells

**Figure 5:**
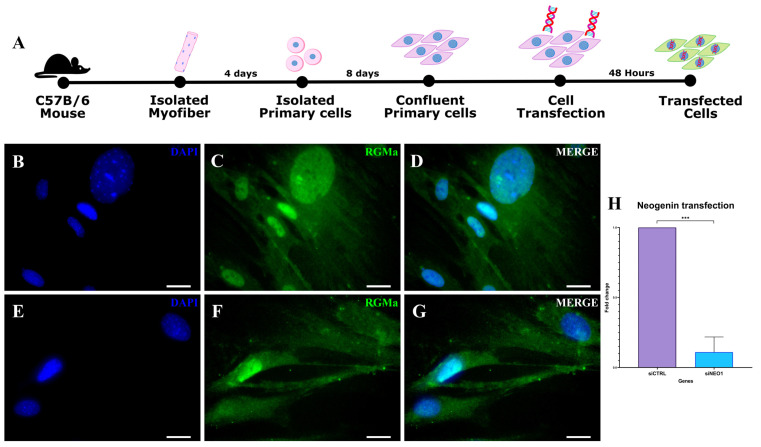
RGMa expression in myoblasts after Neogenin knockdown. (**A**) Timeline of the cell isolation, culturing, and transfection. (**B**–**D**) Primary cells transfected using siRNA-control. (**B**) DAPI nuclear staining (blue); (**C**) RGMa immunofluorescence (green) in cells transfected with the control, showing RGMa nuclear signal. (**D**) Merged image of (**B**,**C**). (**E**–**G**) Primary skeletal muscle cells transfected using siRNA-Neogenin. (**E**) DAPI nuclear staining (blue). (**F**) RGMa immunofluorescence (green) in cells transfected with siRNA-Neogenin, showing the absence of nuclear localization in some cells. (**G**) Merged image of (**E**,**F**). (**H**) qPCR analysis shows efficient knockdown of Neogenin expression compared to control. A statistically significant difference was observed between groups, indicating efficient Neogenin transfection (*** *p* < 0.001). Scale bar: 20 μm.

To investigate whether RGMa nuclear localization depends on Neogenin, we performed Neogenin knockdown in the primary skeletal muscle cells (Figure 5). After 48 h post-transfection (Figure 5A), knockdown efficiency was confirmed by qPCR, showing a significant reduction in Neogenin expression compared to the siRNA-control sample (Figure 5H). Most cells transfected with siRNA-Neogenin lacked nuclear RGMa staining (Figure 5E–G and Supplemental Figure S3), in contrast to the clear nuclear RGMa expression observed in siRNA-control-transfected cells (Figure 5B–D). These results indicate that RGMa nuclear localization in myoblast progeny depends on Neogenin expression.

### 3.4. RGMa Transcripts Are Essential for Skeletal Muscle Cell Viability and Proliferation

**Figure 6:**
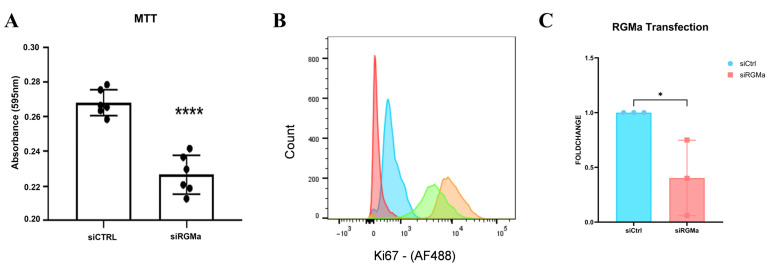
Effects of RGMa knockdown on viability and proliferation of primary skeletal muscle cells. (**A**) Cell viability of these cells, transfected with siRNA-control or siRNA-RGMa, assessed by MTT assay. Asterisks indicate statistically significant differences, **** *p* < 0.0001. (**B**) Representative histograms showing Ki67 fluorescence intensity: negative controls (red and blue curves), siRNA-control (orange), and siRNA-RGMa (green). (**C**) qPCR validation of RGMa knockdown 48 h post-transfection. Data are presented as mean ± SD. *p* < 0.05 (*). Statistical analysis was performed using GraphPad Prism (version 9.5); normality was assessed with the Shapiro–Wilk test, followed by two-tailed Student’s *t*-test. All experiments were performed in triplicate.

To investigate the biological role of RGMa in myoblasts derived from muscle satellite cells, we performed RGMa knockdown using siRNA (siRNA-RGMa). Knockdown efficiency was confirmed by qPCR 48 h after transfection, showing a significant reduction in RGMa transcript levels compared to the siRNA-control group (Figure 6C). Next, we assessed cell viability using the MTT assay (Figure 6A). RGMa knockdown led to a significant reduction in cell viability compared to control cells. To evaluate the impact of RGMa knockdown on cell proliferation, we analyzed Ki67 expression by flow cytometry. After 48 h of siRNA treatment, 95.5% of siRNA-control-transfected cells were Ki67-positive, while only 87% of siRNA-RGMa knockdown cells expressed this proliferation marker (Figure 6B).

### 3.5. RGMa Knockdown Reduces Expression of Myogenic and Osteogenic Markers in Primary Skeletal Muscle Cells

**Figure 7:**
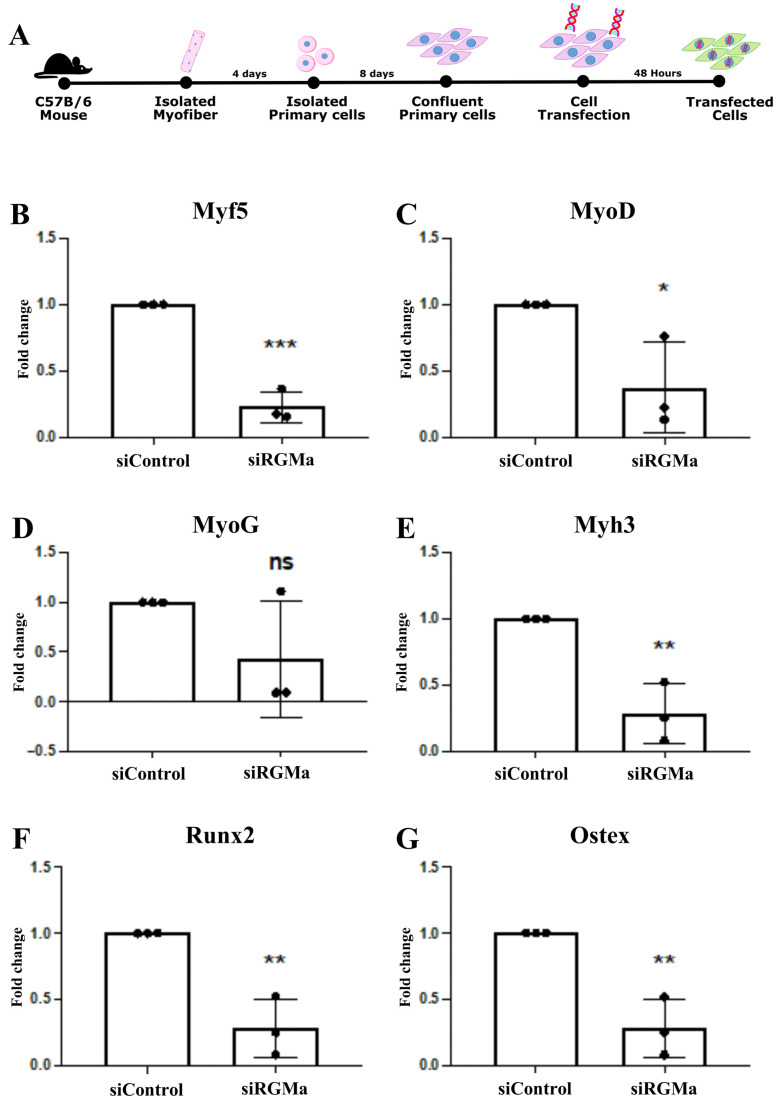
Gene expression analysis of myogenic and osteogenic markers in primary skeletal muscle cells following RGMa knockdown. (**A**) Timeline of the cell isolation, culturing, and transfection. qPCR analysis revealed significant downregulation of myogenic markers (MyoD, Myf5, Myogenin, Myh3) (**B**–**E**) and osteogenic markers (Runx2 and Osterix) (**F**,**G**) in cells transfected with siRNA-RGMa compared to siRNA-control. Data are presented as mean ± SD. *p* < 0.05. A statistically significant difference was observed between groups (* *p* < 0.05, ** *p* < 0.01, *** *p* < 0.001) Statistical analyses were performed using GraphPad Prism software (version 7.0); normality was assessed with the Shapiro–Wilk test, followed by two-tailed Student’s *t*-test.

To investigate whether RGMa knockdown affects the expression of lineage-specific genes, we analyzed the mRNA levels of myogenic markers (Myf5, MyoD, Myog, and Myh3) in primary skeletal muscle cells, using qPCR (Figure 7). Quantitative analysis showed a downregulation of the expression of all analyzed myogenic markers in these cells transfected with siRNA-RGMa relative to the control (Figure 7B–E). Given that these cells also possess osteogenic potential, we evaluated whether the expression of osteogenic markers (Runx2 and Osterix) was affected. Both genes were also downregulated in RGMa-deficient cultures (Figure 7F,G).

## 4. Discussion

RGMa is a glycosylphosphatidylinositol (GPI)-anchored protein originally characterized as a repulsive axon guidance molecule in the developing nervous system. However, growing evidence indicates broader biological functions for RGMa, including roles in non-neuronal tissues such as skeletal muscle. For instance, RGMa has been reported to be expressed in Pax7^+^ pioneer muscle cells in the dermomyotome of chicken somites [5] and to promote both hypertrophy and hyperplasia in C2C12 myoblasts and primary muscle cells [6]. Here, we expand on these findings by characterizing RGMa expression and function in quiescent and activated myosatellite cells and their progeny.

Immunofluorescence performed in sections of skeletal muscle tissue revealed that RGMa colocalizes with Pax7 in quiescent myosatellite cells. RGMa protein could also be localized in all the myonuclei in peripheral cells of isolated myofibers, including in Pax7^+^ cells, consistent with activated myosatellite cells. Interestingly, RGMa was consistently detected in the nuclei of primary skeletal muscle cells maintained in culture for up to eight days. This nuclear localization is unexpected, considering RGMa is typically membrane-bound via its GPI-anchor. Our analysis using PSORTII software confirmed that RGMa lacks a canonical nuclear localization signal (NLS), suggesting it does not translocate to the nucleus independently. This raises the possibility that RGMa reaches the nucleus via an indirect mechanism [13,14].

One such candidate is Neogenin, a RGMa canonical receptor [3,15,16,17] and a known transmembrane protein involved in several signaling pathways. PSORTII predicted a nuclear localization capacity for Neogenin, and in fact, we could observe its predominant localization in the perinuclear region of MuSCs, with occasional nuclear presence. Strikingly, RGMa nuclear localization was markedly reduced upon Neogenin knockdown, supporting the hypothesis that Neogenin may facilitate RGMa entry into the nucleus, potentially via a co-transport mechanism. This raises interesting mechanistic questions about receptor-ligand trafficking in stem cells, a topic that remains poorly understood.

Beyond localization, our functional assays demonstrated that RGMa knockdown significantly impairs the viability and proliferative capacity of primary skeletal muscle cells. The observed reduction in metabolic activity by MTT assay and decreased expression of the proliferation marker Ki67 suggest that RGMa supports cell survival and proliferation in this context. These findings align with previous reports linking RGMa to cellular growth in other systems, like neural stem cells or cancer lines [18,19,20], but expand its relevance to skeletal muscle regeneration. Further functional experiments should be conducted, but using knockout strategies (CRISPR, for example) to completely remove RGMa from skeletal muscle cells, as well as its opposite effect (RGMa overexpression).

Further, RGMa silencing resulted in a broad downregulation of key myogenic markers (Pax7, MyoD, Myf5, Myogenin, and Myh3), indicating impaired commitment and differentiation toward the myogenic lineage. Interestingly, RGMa knockdown also led to reduced expression of the osteogenic transcription factors Runx2 and Osterix, suggesting that the effect of RGMa may not be limited to the muscle lineage but may extend to multipotent fate decisions in MuSCs [20,21]. Given that RGMa can function as a BMP co-receptor [22], and BMP signaling is a well-established modulator of both myogenic and osteogenic programs, it is plausible that RGMa modulates lineage fate through BMP-related pathways. Future studies should explore this intersection in more detail.

Taken together, our findings suggest that RGMa is not only present but functionally important within the nuclei of skeletal muscle cells. The dependency on Neogenin for nuclear localization, combined with the effects of RGMa knockdown on viability, proliferation, and differentiation, opens intriguing possibilities about the non-canonical roles of RGMa in stem cell biology.

## 5. Conclusions

Our findings demonstrate that RGMa is expressed in quiescent and activated myosatellite cells and is localized in the nuclei of their progeny. RGMa nuclear localization is dependent on Neogenin, which likely acts as a co-transporter. Neogenin knockdown significantly reduced RGMa nuclear presence, supporting this mechanistic link. Functionally, RGMa promotes skeletal muscle cell proliferation, as evidenced by decreased Ki67 expression following RGMa knockdown. Additionally, RGMa is essential for maintaining the myogenic transcriptional program in these cells, as its depletion led to marked downregulation of key myogenic markers. These results position RGMa as a critical regulator of skeletal muscle cell function and suggest a novel intracellular role for this classical membrane-associated guidance molecule.

## Figures and Tables

**Table 1:** Analysis of nuclear localization signals (NLS) in RGMa and Neogenin proteins. Prediction of subcellular localization and identification of nuclear localization signal (NLS) peptides performed using PSORTII software WWW version.

Protein	Accession Number	Monopartite		Bipartite	NLS Score	Dileucine Motif Tail	NNC Tendency	
		Pat4	Pat7					
**RGMa**	AAH59072.1	NONE	NONE	NONE	−0.47	LL AT 37’; LL AT 381’	70.60%	Cytoplasmatic
**Neogenin**	AAH54540.1	1 at 1157’ (KKKR)	NONE	NONE	−0.16	NONE	89%	Nuclear

## Data Availability

The data presented in this study are available upon request from the corresponding author, as the complete data cannot be made available due to privacy restrictions.

## Supplementary Materials

The following supporting information can be downloaded at: https://www.mdpi.com/article/10.3390/cells15020161/s1.
