# Supplementary figures and images for: RGMa Nuclear Localization in Skeletal Muscle Cells Reveals a Novel Role in Cell Viability and Proliferation
Source: Cells. 2026 Jan 15;15(2):161. doi: 10.3390/cells15020161 (PMC12839624; doi:10.3390/cells15020161)

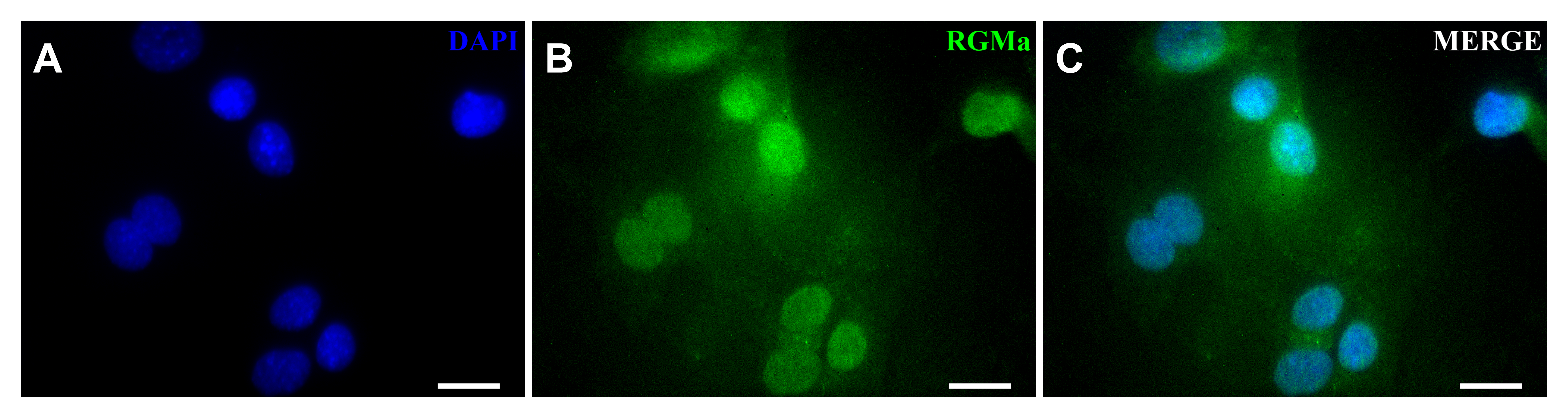

Supplement: Supplementary file 1 [file cells-15-00161-s001.zip › Supplementary figure 1.tif]

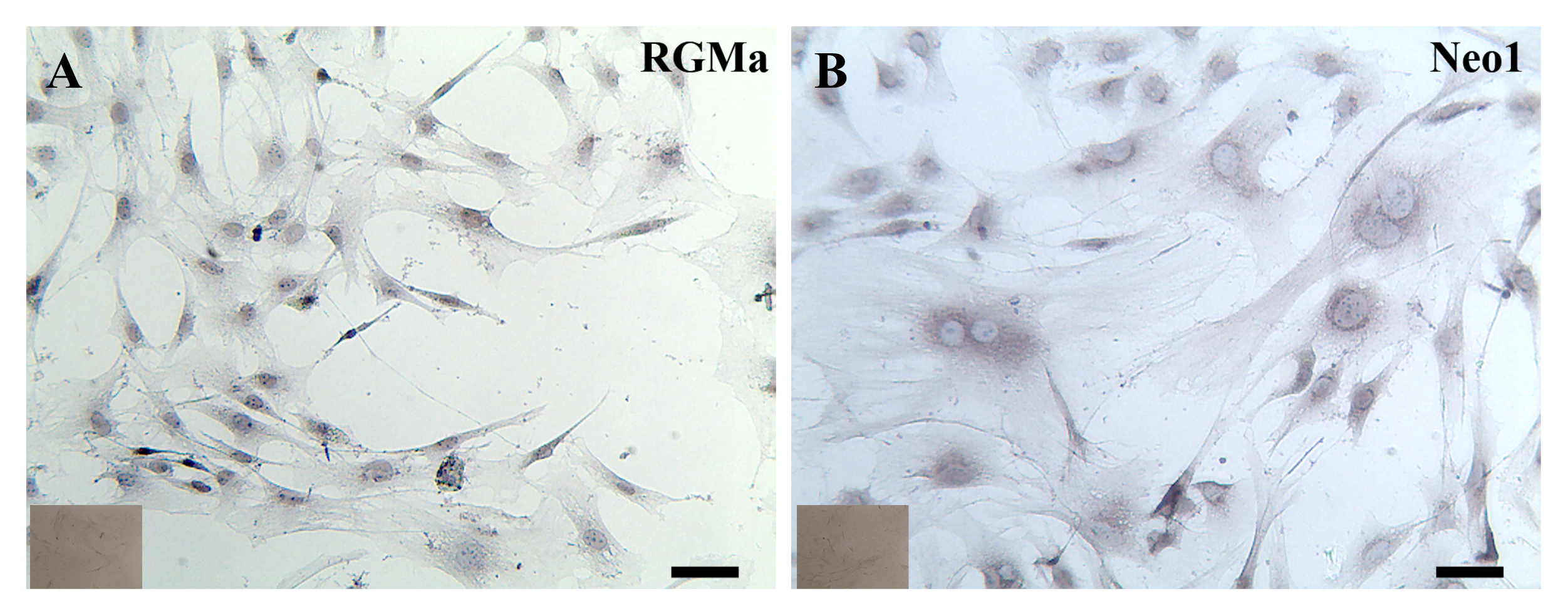

Supplement: Supplementary file 1 [file cells-15-00161-s001.zip › Supplementary figure 2.tif]

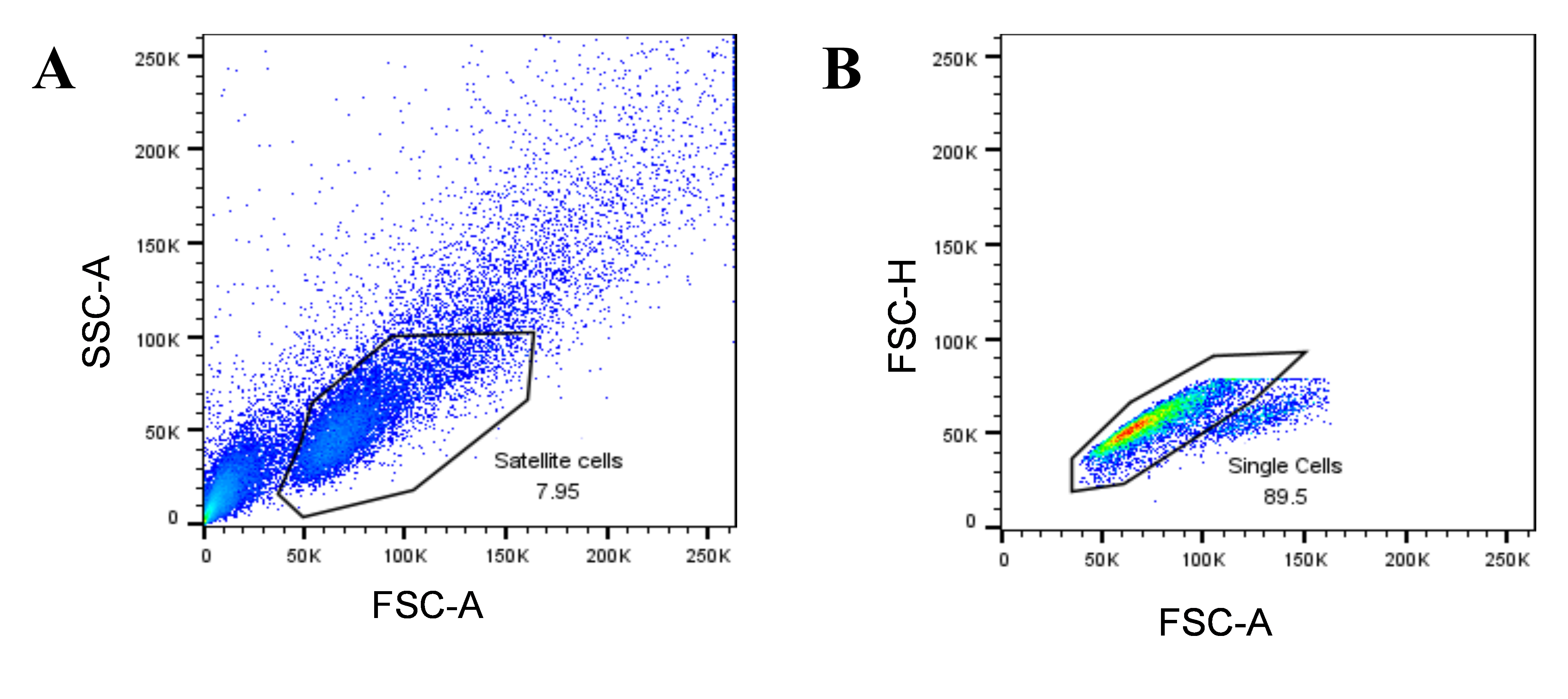

Supplement: Supplementary file 1 [file cells-15-00161-s001.zip › Supplementary figure 4.tif]
